# Abscopal Resolution of a Hepatic Metastasis in a Patient with Metastatic Cholangiocarcinoma Following Radical Stereotactic Body Radiotherapy to a Synchronous Early Stage Non-small Cell Lung Cancer

**DOI:** 10.7759/cureus.4082

**Published:** 2019-02-16

**Authors:** Julian O Kim, Christina A Kim

**Affiliations:** 1 Radiation Oncology, Cancer Care Manitoba, University of Manitoba, Winnipeg, CAN; 2 Oncology and Hematology, CancerCare Manitoba, University of Manitoba, Winnipeg, CAN

**Keywords:** stereotactic radiotherapy, abscopal effect, cholangiocarcinoma, non-small cell lung cancer, liver metastasis

## Abstract

This case report describes the abscopal resolution of a liver metastasis in a patient with two separate primary malignancies. A 70-year-old male with an unresectable cholangiocarcinoma with an associated 5 cm liver metastasis was found during his staging investigations to have a 1.8 cm right upper lobe lung tumor. A CT-guided biopsy of the lung tumor revealed a primary adenocarcinoma of lung origin. Given the expected worse prognosis of the metastatic cholangiocarcinoma, after review of his case in provincial gastrointestinal and lung tumor boards, he was treated with eight cycles of palliative gemcitabine and cisplatin chemotherapy. Post eight cycles, the disease in the liver and the lung was stable. After completion of first line palliative systemic therapy, radical stereotactic body radiotherapy (SBRT), consisting of 48 Gy in four fractions, was delivered to the right upper lobe non-small cell lung cancer (NSCLC) primary. Three months post-completion of the SBRT, restaging CT scans were performed which revealed the intriguing spontaneous and complete resolution of his liver metastasis. These findings were confirmed on subsequent MRI imaging of his liver. As his liver metastasis was well outside of the SBRT fields, the spontaneous resolution of his liver metastasis presents clinical evidence of the abscopal effect of cholangiocarcinoma in response to SBRT to his lung tumor.

## Introduction

The spontaneous regression of an out-of-field tumor following radiotherapeutic treatment to a separate tumor nodule is a rare and intriguing phenomenon [1]. This phenomenon, known as the abscopal effect, was first described by Mole in 1953 [2]. Radiotherapy, especially the hypofractionated doses of radiotherapy commonly used in stereotactic body radiotherapy (SBRT) [3], has been observed to serve as a trigger for the abscopal effect [4]; however, this is a relatively rare phenomenon seen during routine clinical care. We present the case of an abscopal resolution of a liver metastasis related to a cholangiocarcinoma in response to out-of-field SBRT to a separate NSCLC primary.

## Case presentation

**Figure 1:**
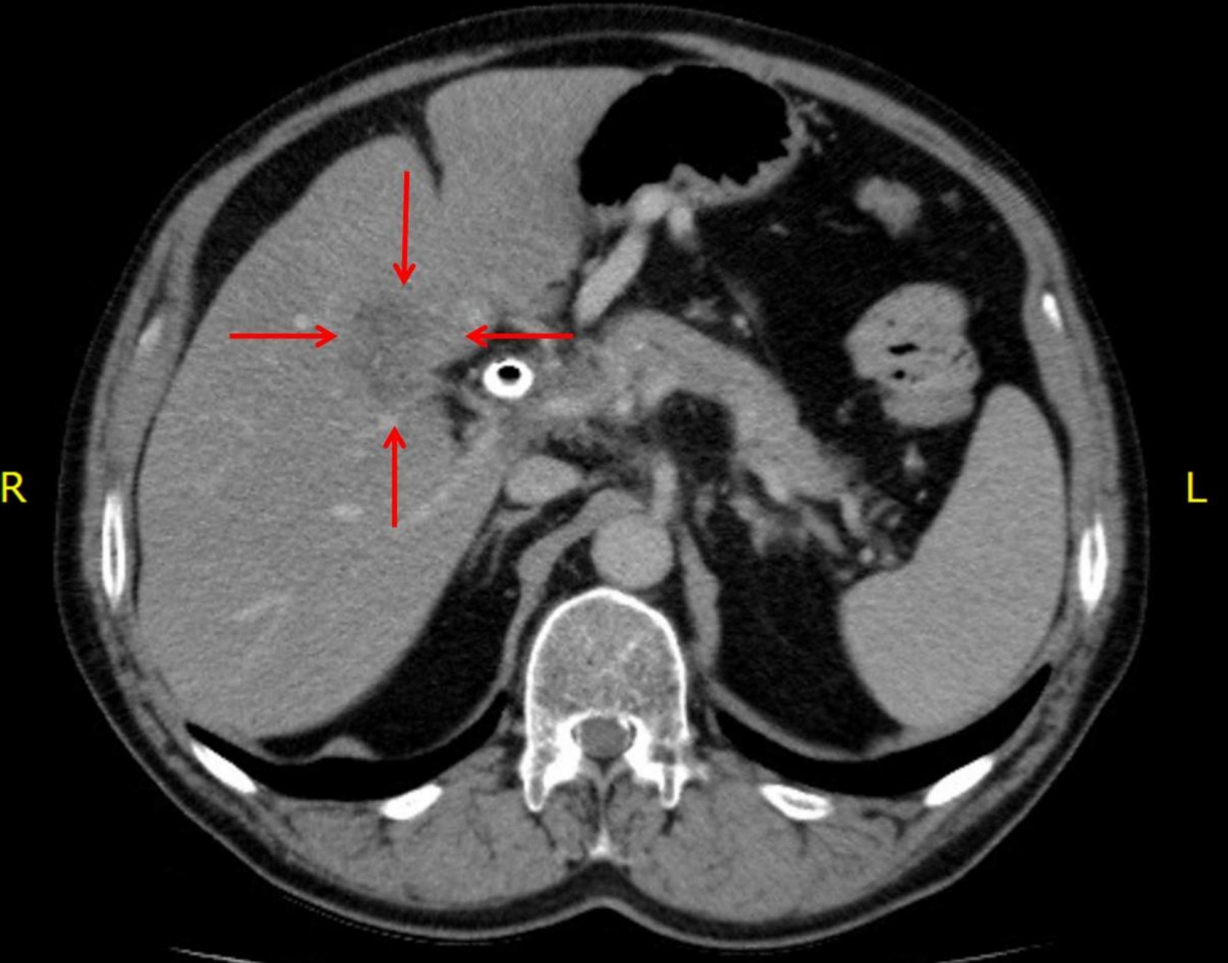
Post-chemotherapy, pre-SBRT CT scan of the abdomen demonstrating a 5.4 cm x 2.8 cm liver metastasis of the cholangiocarcinoma. SBRT, stereotactic body radiotherapy.

A 70-year-old male presented to his primary care physician with jaundice. Bloodwork revealed a bilirubin of >100 µmol/L. A CT scan of the abdomen and pelvis revealed moderate intrahepatic biliary dilatation and a stricture of the common hepatic duct within the head of the pancreas. Soft tissue infiltration around the common hepatic artery and portal vein was suspicious for a cholangiocarcinoma. On subsequent imaging, an ill-defined hypoattenuating mass (5.4 cm x 2.8 cm) was observed adjacent to the hepatobiliary tract extending into the right lobe of the liver consistent with a liver metastasis from the cholangiocarcinoma (Figure 1). He underwent endoscopic retrograde cholangio-pancreatography (ERCP) and bile duct brushings revealed adenocarcinoma cells. Functionally, he was well with an Eastern Cooperative Oncology Group (ECOG) performance status of 1.

His previous medical history was remarkable for gout, hypothyroidism, dyslipidemia, benign prostatic hypertrophy, appendectomy, and remote pancreatitis. His medications included levothyroxine, allopurinol, omeprazole, rosuvastatin, and vitamin B12. He had a 30 pack year history of smoking, and quit 19 years ago. At baseline, he consumed two to three alcoholic drinks per day but has abstained from alcohol since the time of his diagnosis.

**Figure 2:**
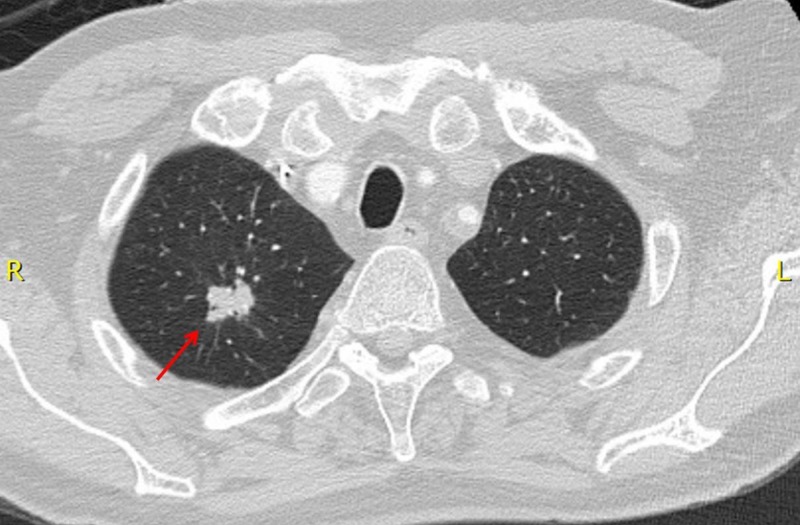
CT chest, pre-SBRT, demonstrating the spiculated 1.8 cm adenocarcinoma (NSCLC) of the right upper lobe of the lung. NSCLC, non-small cell lung cancer.

As part of his initial staging investigations, a CT scan of the chest was performed which revealed a 1.8 cm spiculated right apical pulmonary nodule (Figure 2). A transthoracic, image guided biopsy of the pulmonary nodule revealed an adenocarcinoma. Immunohistochemistry (IHC) was positive for cytoketatin 7 (CK7), thyroid transcription factor 1 (TTF-1) and Napsin A, and negative for cytokeratin 20 (CK20), consistent with a primary NSCLC. IHC for anaplastic lymphoma kinase (ALK) was negative and programmed death-ligand 1 (PD-L1) was 1% to 49%. There were insufficient cells in the bile duct brushings to do mismatch repair (MMR) testing or to compare the NSCLC and biliary tract specimens in terms of morphology and IHC profile. However, because the lung tumor was small in size, with no evidence of hilar or mediastinal lymphadenopathy, these were deemed to represent two distinct primary cancers.

After review of his case in both the lung and gastrointestinal provincial tumor boards, he received eight cycles of palliative-intent cisplatin and gemcitabine chemotherapy. He required a dose reduction because of rash and neutropenia. During chemotherapy, the liver metastasis grew slightly to 5.4 cm x 3.6 cm and appeared more conspicuous compared to a prior examination. The lung mass, however, remained stable, as per response evaluation criteria in solid tumors (RECIST criteria) [5]. Chemotherapy was stopped and the patient continued on observation. On a follow-up CT scan, both the disease in the chest and the abdomen remained stable. As the patient maintained an excellent functional status over one year since the initial diagnosis, the option of SBRT to the lung lesion was considered. 

**Figure 3:**
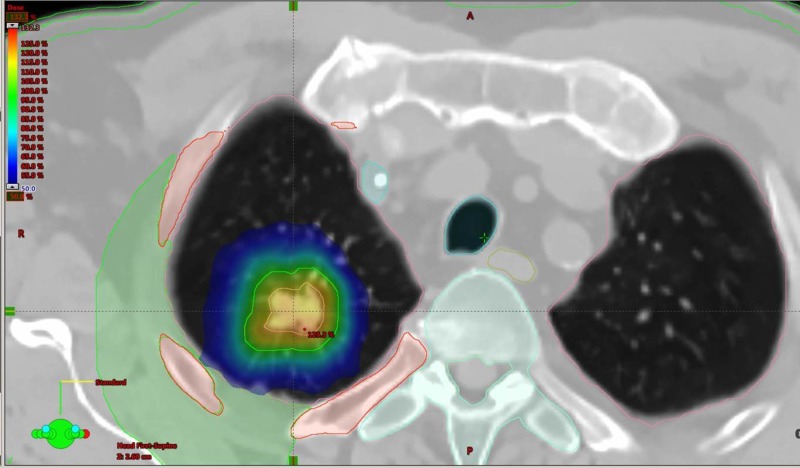
SBRT (48 Gy in four fractions) treatment volumes and dose distributions for the right upper lobe adenocarcinoma (NSCLC) in the axial plane.

**Figure 4:**
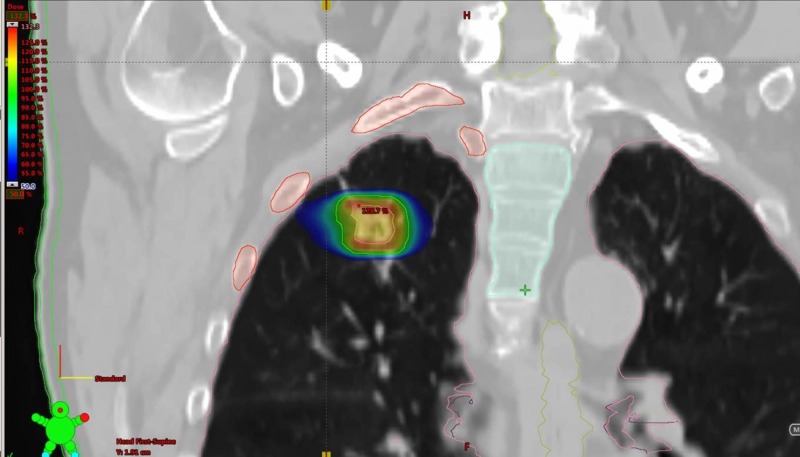
SBRT (48 Gy in four fractions) treatment volumes and dose distributions for the right upper lobe adenocarcinoma (NSCLC) in the coronal plane.

As part of the pre-SBRT assessments a positron emission tomography (PET) scan and CT brain were performed, which confirmed that there were no other sites of distant or nodal metastatic disease consistent with an AJCC 8th ed. [6] stage of T1bN0M0 NSCLC. He was then treated with radical SBRT to the right upper lobe NSCLC with a total dose of 48 Gy in four fractions (Figures 3-4). His treatment was planned using a four-dimensional CT simulation scan with fusion of a pre-treatment PET scan to aide with delineation of the primary tumor. His SBRT was delivered using two 240-degree RapidArc™ 6 megavoltage photon arcs using a Varian Edge Linear Accelerator. Daily cone beam CT scans were used for purposes of daily image guidance for his SBRT. He tolerated his SBRT very well and did not suffer any acute severe adverse effects as a result of treatment.

**Figure 5:**
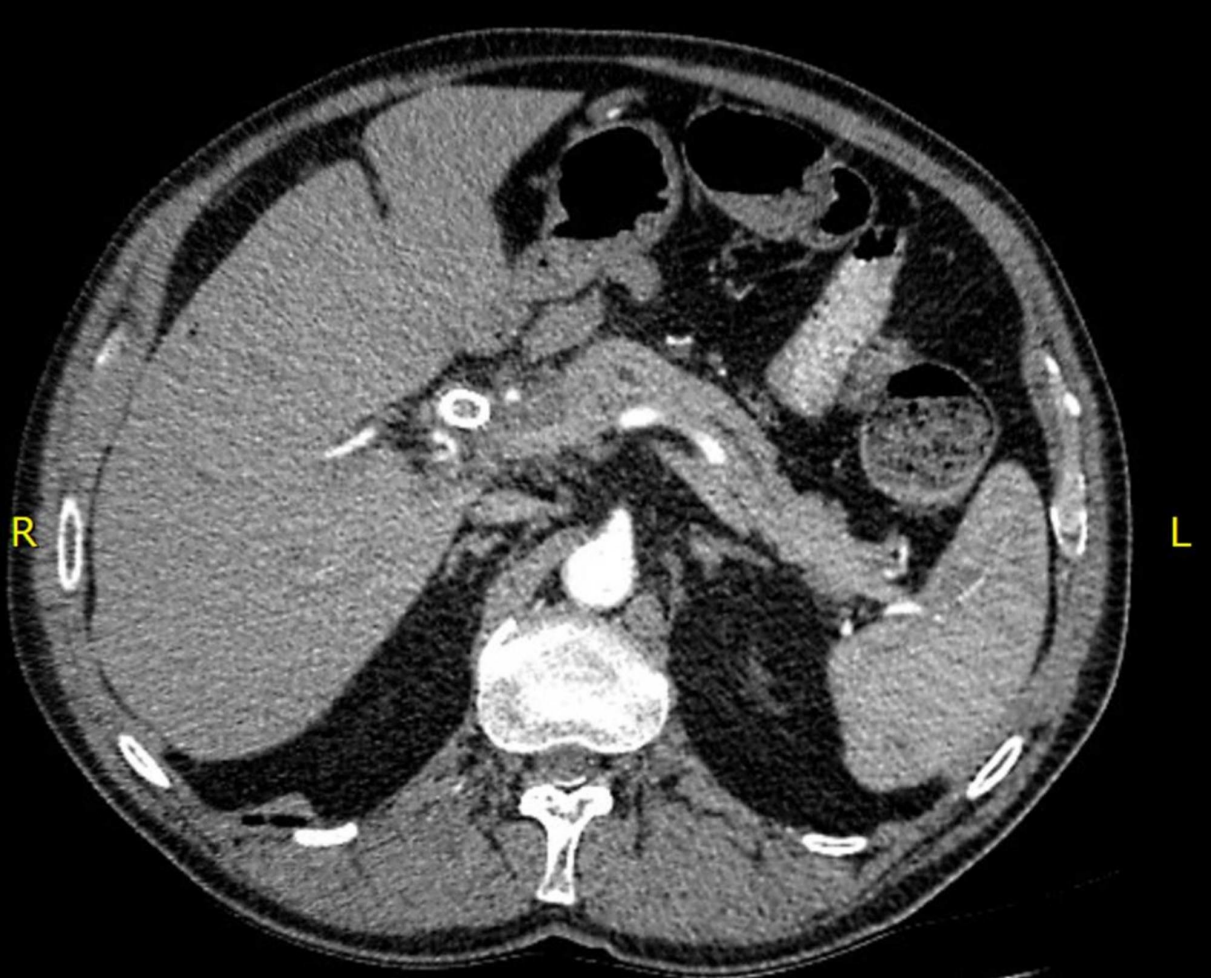
CT scan of the abdomen from three months post-SBRT to the right upper lobe lung adenocarcinoma (NSCLC) demonstrating the complete resolution of the liver metastasis from the cholangiocarcinoma.

**Figure 6:**
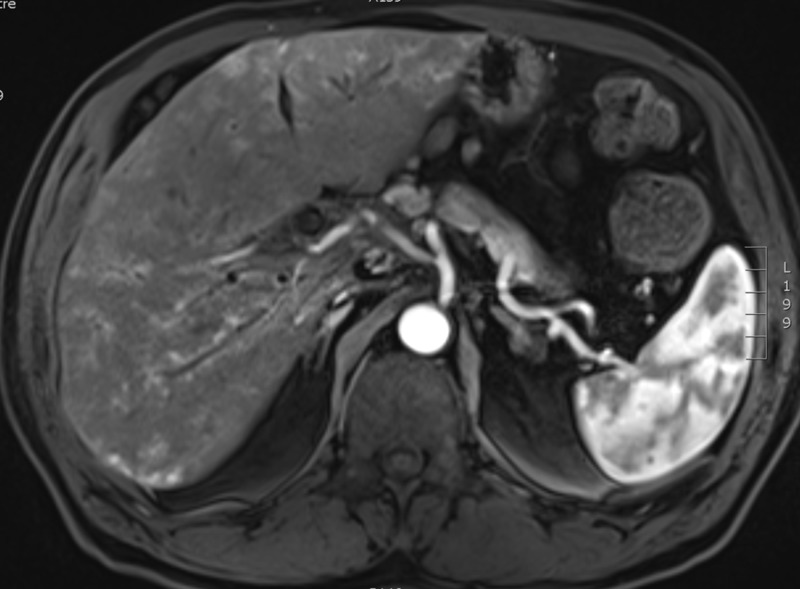
Arterial phase MRI liver (VIBE pulse sequence) obtained four months post-SBRT to the NSCLC demonstrating the complete resolution of the hepatic metastasis. VIBE, volumetric interpolated breath-hold examination.

**Figure 7:**
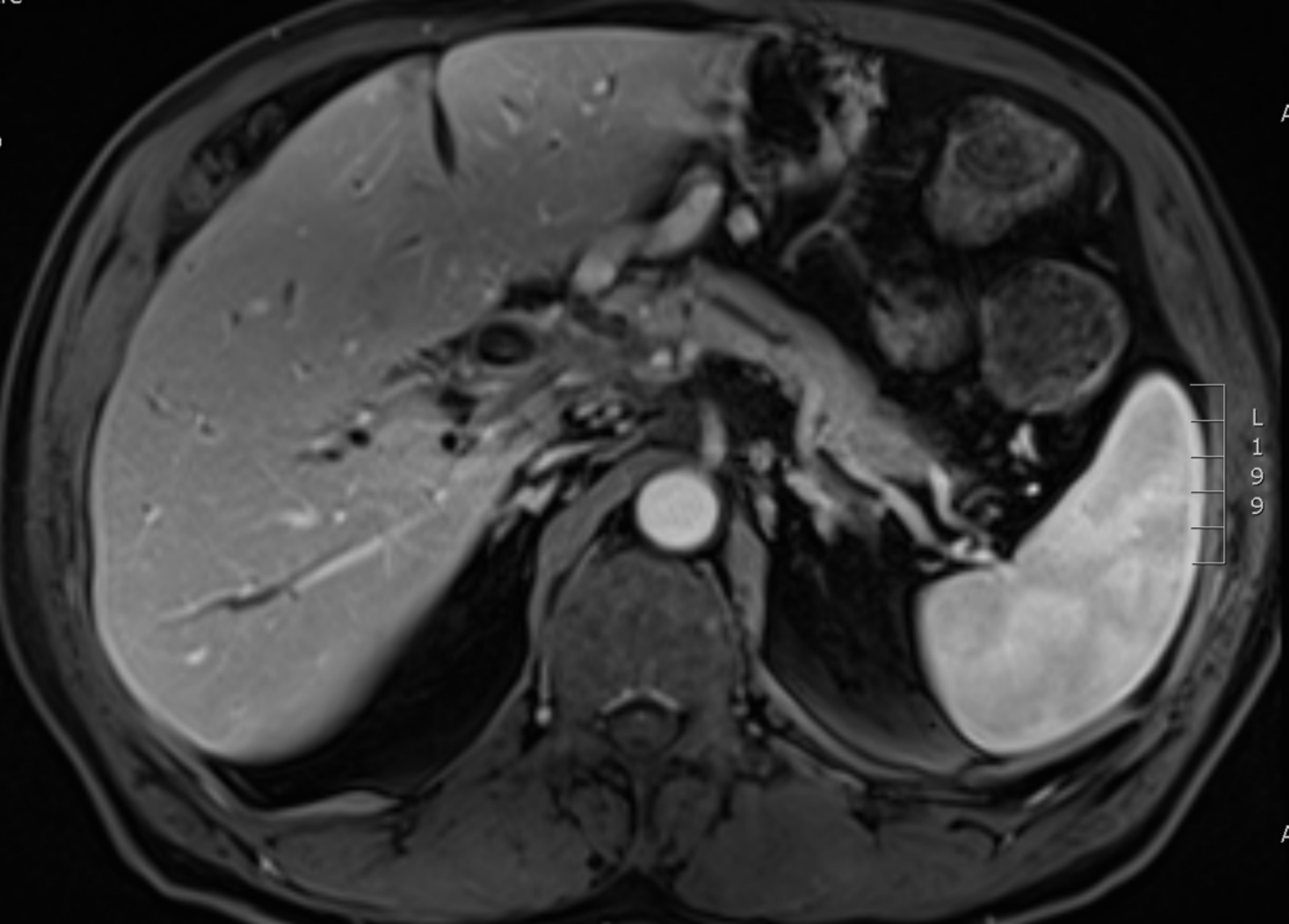
Venous phase MRI liver (VIBE sequence) obtained four months post-SBRT to the NSCLC demonstrating the complete resolution of the hepatic metastasis.

Three months post-completion of SBRT to the NSCLC, he presented to his primary care physician with a jaundiced appearance and mild scleral icterus. Bloodwork revealed transaminases and cholestatic liver enzymes were three to five times the upper limit of normal. Total bilirubin was 79 µmol/L, and direct bilirubin was 61 µmol/L. A CT scan of the chest, abdomen, and pelvis was performed in order to rule out tumor progression as a cause for his change in clinical status. The CT scan of the chest revealed stability of the right upper lobe lung tumor. Within the abdomen, the hepatic metastasis had completely resolved (Figure 5). Bloodwork repeated one week later showed that his liver enzymes and bilirubin had completely normalized. The carbohydrate antigen 19-9 (CA 19-9) had decreased from 41 to 14 (upper limit of normal = 34 U/mL). A dedicated MRI of the liver was performed four months post-completion of the SBRT to the NSCLC in order to further assess the status of the liver metastasis. This scan confirmed the complete and spontaneous out-of-field resolution of the hepatic metastasis in keeping with an abscopal event (Figures 6-7).

The median survival of patients with locally advanced or metastatic cholangiocarcinoma treated with palliative gemcitabine/cisplatin chemotherapy is 11.7 months [7]. This patient is now 21 months removed from his initial diagnosis of unresectable cholangiocarcinoma and he is enjoying a more protracted survival than expected. He remains functionally active, exercising on a daily basis and is able to maintain employment as the proprietor of a construction company. It is unclear why the abscopal effect was limited to a metastasis in this case, however, it is plausible that the primary cholangiocarcinoma tumor may also change with time and further follow-up will be performed to monitor for this possibility.

## Discussion

The abscopal effect is a rare and poorly understood phenomenon. Although the precise mechanism of action of the abscopal effect has yet to be completely elucidated, preclinical studies point towards a complex interplay between the initially targeted tumor, the radiotherapy, the tumor microenvironment, and the host immune system resulting in an acquired antitumoral immunity. 

The various facets of this acquired post-radiotherapy antitumoral immunity, the cornerstone of the abscopal effect, have been pieced together via bench and animal model experiments. Several cancer cell lines have been demonstrated to alter their gene expression profiles after radiotherapy in such a way as to increase their susceptibility to be killed by cytotoxic T-cells [8]. In other studies, radiotherapy has been observed to alter the irradiated tumor in such a way as to increase tumor antigen presentation by dendritic cells, thereby resulting in a process of autovaccination of the host against other distant tumors [9]. In murine models, this autovaccination effect following radiotherapy has been demonstrated to increase T-cell mediated antitumoral immune responses [10], ultimately resulting in enhanced tumor cell death. Further corroborating data which implicate changes in cell-mediated immunity to the abscopal effect come from studies focused on the behavior of programmed cell death protein-1 (PD-1) expression following SBRT. In one study, PD-1 blockade plus SBRT in mice with renal cell carcinoma or melanoma tumor xenographs led to near complete tumor regression [11]. By contrast, PD-1 expressing (wild type) mice treated with SBRT did not exhibit any induced abscopal effect. Finally, the p53 protein complex, which has been implicated in several important components of cytotoxic T-cell related tumor cell deaths, has been observed to play a mediating role of the abscopal effect following SBRT type doses of radiotherapy to primary tumors in mice models [3] thereby adding plausibility to the acquired antitumoral immune response hypothesis to explain the mechanism of the abscopal effect.

Immune dysregulation has been implicated in the carcinogenesis of a number of cancers, and is supported by reports of spontaneous tumor regression, thought to be driven by the body’s immune response to the tumor. Spontaneous tumor regression has been reported in cancers of hepatobiliary origin, including pure hepatocellular carcinoma (HCC) and mixed HCC/intrahepatic cholangiocarcinoma [12]. There have also been reports of abscopal regression of HCC after radiation of distant bony metastatic disease [13]. Such cases support the role of drugs which target the immune system [14], and there is now a role for the anti-PD-1 antibody, nivolumab, in the management of advanced HCC [15]. The role of the immune system in pure cholangiocarcinoma is less clear, however, there are rare case reports of spontaneous regression [16]. To the best of our knowledge, there have been no cases reported in the literature of spontaneous regression of pure cholangiocarcinoma tumors following radical SBRT to an out-of-field synchronous primary tumor.

Overall, the role of immunotherapy in gastrointestinal tumors is limited, with the exception of mismatch repair deficient (dMMR) or microsatellite instability high (MSI-H) tumors. In a study of 10 patients with dMMR colorectal cancer, 40% had a response to the PD-L-1 antibody pembrolizumab [17]. In an expanded study [18] evaluating the effect of pembrolizumab in different types of dMMR tumors, there was an impressive objective response rate of 53% and complete response rate of 21%; however, only four of the 86 patients in this study had a cholangiocarcinoma, three of whom experienced stable disease, and one of whom had a complete response. The FDA has since approved the use of pembrolizumab in dMMR/MSI-H solid tumors that have progressed on prior treatment. This is a potential therapeutic option for patients with refractory dMMR/MSI-H cholangiocarcinoma, however, it should be noted that only approximately 3% of cholangiocarcinomas are dMMR or MSI-H. 

Because of the impact of radiation on the tumor immune environment, studies are emerging which assess the role of radiation in conjunction with immunotherapy. In a phase I study of 35 patients, one of whom had a cholangiocarcinoma, SBRT was given either concurrently or sequentially with ipilumumab, a lymphocyte-associated antigen 4 (CTLA-4) inhibitor. Ten percent of patients experienced a partial response outside of the radiation field [19]. This particular patient with cholangiocarcinoma did not achieve a radiologic response. Another phase 1 study was conducted to assess the safety of pembrolizumab in patients with metastatic solid tumors receiving SBRT [20]. In this study, six of 73 patients assessed had a cholangiocarcinoma. Therapy was well tolerated, and 26.9% of patients experienced an abscopal response, defined as a 30% reduction in a distant, nonirradiated metastasis. Further clinical trials are needed to determine the efficacy of immunotherapy in advanced cholangiocarcinoma, and unleashing the abscopal effect with radiation should be considered in future clinical trials.

## Conclusions

This is the first case report, to the best of our knowledge, of an abscopal effect in a patient with cholangiocarcinoma. Our patient experienced the resolution of a cholangiocarcinoma liver metastasis following SBRT to an unrelated synchronous NSCLC, suggesting that the abscopal effect may not be explicitly tumor specific in nature, and that tumor cell epitopes common to adenocarcinomas arising from different primary tissue types may possibly be responsible for eliciting the abscopal effect *in vivo*.
